# Scanning probe microscopy for rheological analysis of biomolecular condensates

**DOI:** 10.1016/j.xpro.2025.104170

**Published:** 2025-11-06

**Authors:** Aida Naghilou, Alireza Mashaghi

**Affiliations:** 1 Medical Systems Biophysics and Bioengineering, Leiden Academic Centre for Drug Research, Faculty of Science, Leiden University, Einsteinweg 55, 2333 CC Leiden, the Netherlands; 2 Laboratory for Interdisciplinary Medical Innovations, Centre for Interdisciplinary Genome Research, Leiden University, Einsteinweg 55, 2333 CC Leiden, the Netherlands; 3 Research Laboratory of the Department of Plastic, Reconstructive and Aesthetic Surgery, Medical University of Vienna, Spitalgasse 23, 1090 Vienna, Austria

**Keywords:** atomic force microscopy, AFM, Biotechnology and bioengineering, material sciences

## Abstract

Biomolecular condensates are membrane-less phase-separated assemblies that play key roles in cellular organization. Here, we present a protocol to characterize the full range of rheological properties of condensate droplets, from liquid to solid states, using scanning probe microscopy. We describe steps for preparing condensates, cantilever calibration, and condensate measurement. We then detail analysis procedures required to derive the complex shear modulus.

For complete details on the use and execution of this protocol, please refer to Naghilou et al.^1^

## Highlights

• Protocol for measuring condensate rheology via scanning probe microscopy
• Steps for condensate preparation, cantilever calibration, and rheology analysis
• Data acquisition and analysis for viscoelasticity from force-distance curves

## Abstract (editor highlights)

Publisher’s note: Undertaking any experimental protocol requires adherence to local institutional guidelines for laboratory safety and ethics.

## Graphical abstract

**Figure:**
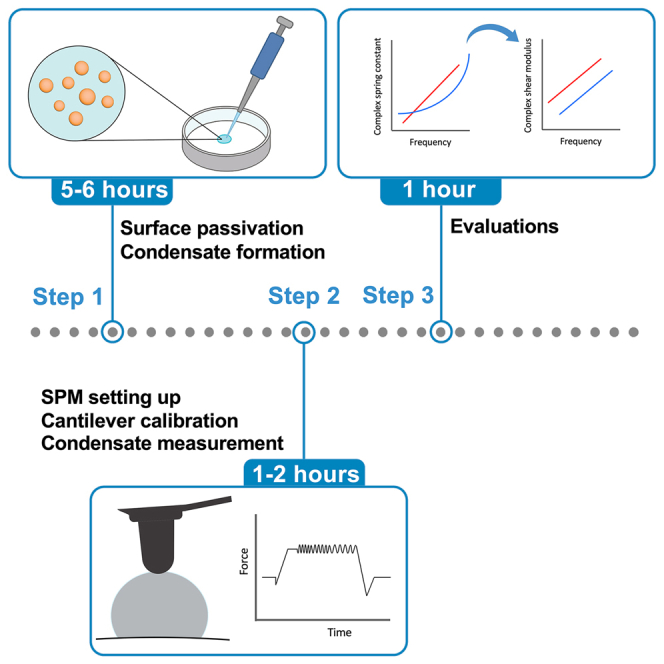

## Teaser

Biomolecular condensates are membrane-less phase-separated assemblies that play key roles in cellular organization. Here, we present a protocol to characterize the full range of rheological properties of condensates, from liquid to solid states, using scanning probe microscopy. We describe steps for preparing condensates, cantilever calibration, and condensate measurement. We then detail analysis procedures required to derive the complex shear modulus.

## Before you begin

The protocol presented here details the necessary procedures for surface passivation, condensate formation, condensate probing with scanning probe microscopy (SPM), as well as evaluations. This protocol is outlined for poly-L-lysine and heparin condensates as they can be made of commercially available reagents, are simplistic in preparation^2^^,^^3^^,^^4^ and allow easy induction of solidification.^1^ The condensates are measured with a JPK CellHesion 200 (Bruker, Germany). The protocol can be applied to characterize droplets with mechanical properties ranging from liquid-like to solid-like. When applying this protocol to condensates other than poly-L-lysine and heparin, it is important to select a cantilever with stiffness appropriate for the mechanical properties of the condensates, use a probe smaller than the condensate diameter, and carefully choose both the dish and cantilever coatings to prevent displacement of the condensate during measurement and to minimize undesired interactions between the condensate and the cantilever, respectively.

### Innovation

Understanding the mechanical properties of biomolecular condensates across their different states from liquid to solid poses significant experimental and technological challenges.^5^ Commonly used methods in condensate studies either offer limited force range, require labeling with fluorescent markers, or are restricted to specific mechanical regimes. Our SPM-based methodology overcomes many of these limitations by enabling comprehensive, label-free rheological characterization on a single platform.

While optical tweezers are routinely used to measure condensate properties, they are constrained to low-force regimes.^6^ In contrast, SPM provides substantially higher forces, enabling the study of transitions from liquid-like to gelled or fibrillar states. Fluorescence recovery after photobleaching is a powerful tool for probing molecular mobility, but is inherently limited to bulk mechanical determination and is subject to label-induced artifacts.^7^^,^^8^ In contrast, our approach directly quantifies both bulk and interfacial mechanical properties without the need for fluorescent labels. SPM also addresses a critical limitation of aspiration-based techniques, which rely on assumptions of Newtonian behavior and fail to measure condensates after fibrillation.^9^ Furthermore, although few studies have applied atomic force microscopy to biomolecular condensates, they have been restricted to probing capillary bridges.^7^^,^^10^ In our method, we can extract frequency-dependent shear moduli of condensates, and capture both surface and bulk mechanics. Beyond these advantages to other techniques, SPM supports high-throughput data acquisition and nanoscopic spatial resolution, making it ideally suited for monitoring dynamic phase transitions in the field of condensate biology.

### Preparation of cantilever insertion

**Timing: 5–10 min**

To simplify passivation, the cantilever can be coated while mounted directly on the SPM head. Follow these steps to insert the cantilever.

**Figure 1:**
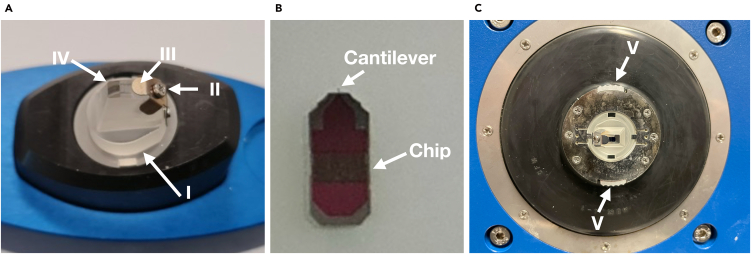
Experimental setup of scanning probe microscope (A) Image of the glass cantilever holder place on a mounting tool. (B) Image of the cantilever. (C) Image of the setup of the cantilever inserted in the glass holder on the scanning probe microscope’s head.

1. Place the glass cantilever holder (denoted as I in Figure 1A) on the mounting tool and loosen the small screw on top (denoted as II in Figure 1A).
2. Using tweezers, grab the cantilever by the chip portion (Figure 1B) and position it beneath the metal clamp of the cantilever holder (denoted as III in Figure 1A).
3. Ensure the chip is securely placed under the metal part, with the cantilever itself aligned on the polished glass section of the holder (denoted as IV in Figure 1A).
4. Carefully adjust to straighten the cantilever under the metal clamp and tighten the screw.
5. Insert the glass holder into the SPM head by pressing it into place, rotating it 90°, and securing the locking tabs (denoted as V in Figure 1C).

### Preparation of surface passivation

**Timing: 5–6 h**

Surface passivation is crucial to minimize condensate adhesion. This section explains the necessary steps for passivating dishes and the cantilever.

6. Slides.
  a. Prepare a fresh 10 mg/mL aqueous bovine serum albumin (BSA) solution in deionized water (dH_2_O).
  b. Incubate surface with copious amounts of BSA to fully cover the surface.
  c. For polystyrene dishes, directly incubate with BSA solution for 30 min, make sure the whole surface is well-covered.
  d. For glass dishes firstly etch silica with 1 M sodium hydroxide for 1 h, wash with dH_2_O, and then incubate with BSA solution for 30 min.^11^
  e. Thoroughly wash the passivated surfaces 5–6 times with copious amounts of dH_2_O.
  f. Let the surfaces to completely dry for at least 4 h.

***Note:*** It is recommended to use the passivated surfaces within 48 h.

**CRITICAL:** Sodium hydroxide is corrosive. Make sure to perform the experiments under a fume hood and wear personal protective equipment.

7. Cantilever.
  a. We used a SAA-SPH-5UM silicon nitride cantilever for poly-L-lysine and heparin condensates.
  b. Prepare a fresh 1% (W/V) aqueous Pluronic F127 solution (PF).
  c. Fill a dish suitable for the SPM measurements (e.g., a culture dish from TPP) with the PF solution.
  d. Using a pipette, withdraw 5 μL from the PF solution.
  e. Gently dispense the droplet until it is hanging from the pipette tip, then place it onto the cantilever without touching the cantilever itself with the tip.
  f. Move the condenser part of the microscope back to allow enough space for placing the SPM head.
  g. Make sure you have enough distance between the cantilever and the dish and place the SPM head on the microscope, thereby immersing the cantilever in the solution.
  h. Incubate cantilever with PF solution for 30 min.
  i. Remove the head and rinse the cantilever by carefully flushing it with dH_2_O to remove excess PF. Take care not to damage the cantilever with the pipette tip while washing.
  j. Place the head in a new dish filled with copious amounts of dH_2_O for 30 min to remove any remaining PF.

***Note:*** The addition of 5 μL drop on cantilever prevents the introduction of air bubbles when immersing the cantilever into the liquid.

***Note:*** Alternatively, the cantilever may be coated prior to insertion into the SPM head, but this increases the risk of breaking it.

***Note:*** Once in place, the cantilever can be reused for multiple experiments. Regular re-passivation is recommended to maintain optimal performance.

### Preparation of condensates

**Timing: approximately 2 h**

8. Prepare the droplets by adding the chemicals on BSA coated dishes or slides in order to reach the following end concentrations:

**Table:** 

Reagent	Final concentration	Amount
Potassium chloride (4 M)	1 M	10 μL
Imidazole (100 mM)	10 mM	4 μL
Sodium azide (0.5% W/V)	0.01% W/V	0.8 μL
Ficoll PM 70 (100 g/L)	50 g/L	20 μL
Heparin (1 M)	40 μM	1.6 μL
Poly-L-lysine (1.4 M)	40 μM	1.14 μL
dH_2_O	N/A	2.46 μL
**Total**	**N/A**	**40 μL**

***Note:*** Potassium chloride is used to modulate electrostatic interactions, imidazole serves as a buffer to maintain stable pH and charge states of the components, and sodium azide is added to prevent microbial growth (optional).

***Note:*** It is recommended to prepare a working buffer containing potassium chloride, imidazole, sodium azide, and Ficoll PM 70 prior to use. Thereafter, gently mix in the heparin, followed by the gentle addition and mixing of poly-L-lysine. A subtle change in the solution’s appearance to a milky suspension is visible by the naked eye.

**CRITICAL:** Sodium azide is highly toxic, if you opt to use it, make sure to wear appropriate personal protective equipment and perform the experiments under a fume hood.

**CRITICAL:** Allow the droplets to settle on the dish surface before SPM measurements for approximately 2 h until most coalescence events cease.

**CRITICAL:** A sample volume of at least 40 μL is recommended.

## Key resources table

**Table:** 

REAGENT or RESOURCE	SOURCE	IDENTIFIER
**Chemicals, peptides, and recombinant proteins**		

Bovine serum albumin	Sigma-Aldrich	A2153
Potassium chloride	Sigma-Aldrich	P9333
Imidazole	Sigma-Aldrich	56750
Sodium azide	Sigma-Aldrich	8.22335
Ficoll PM 70	Sigma-Aldrich	F2878
Heparin sodium salt from porcine intestinal mucosa	Sigma-Aldrich	H3393
Poly-L-lysine hydrochloride	Sigma-Aldrich	P2658
Pluronic F-127	Sigma-Aldrich	P2443

**Other**		

Silicon nitride cantilever	Bruker	SAA-SPH-5UM
JPK CellHesion 200	Bruker	N/A
Culture dish	TPP	93040
Evaluation notebook	Primary research article^1^	https://doi.org/10.5281/zenodo.14500842

## Step-by-step method details

### SPM measurement

**Timing: approximately 2 h**

This step describes how to prepare the SPM setup for measurements, calibrate the cantilever and its phase lag for further use in evaluation procedures, and how to perform measurements on condensates. We used a JPK CellHesion 200 (Bruker, Germany) equipped with a Ti2 microscope (Nikon, Japan) delivering real-time *in situ* images of measurements by means of a 20×/0.4 objective.

1. Head placement.
  a. Place the dish containing 40 μL bulk volume of settled droplets in the sample holder on the microscope stage.
  b. Move the condenser back to allow enough space for placing the SPM head.
  c. Using a pipette, carefully withdraw 5 μL from the supernatant of the condensates.
  d. Gently dispense the droplet until it is hanging from the pipette tip, then place it onto the cantilever without touching the cantilever itself with the tip.
  e. Place the SPM head on the microscope, allowing for the insertion of the cantilever in the bulk drop, generating a capillary and setting the cantilever under the liquid.
2. Setting up the SPM.
  a. Turn on the SPM machine at least 30 min before performing the experiments.
  b. Turn on the JPK controller module by pressing the rear power button, followed by the front button.
  c. Power on the connected computer and log in.
  d. Turn on the microscope X-Y stage.
  e. Launch the JPK Control software (JPK SPM Desktop).
  f. Select Microrheology mode in the “Choose Experiment” tab.
  g. In the “Setup Experiment” tab, turn on the laser.
  h. Adjust the two alignment screws on the right and back-right side of the SPM head to direct the laser beam onto the cantilever. Monitor the sum signal and optimize it to its maximum voltage.
  i. Adjust the left and back-left screws to center the laser on the photodiodes, aiming for zero vertical and lateral deflection.

**CRITICAL:** Wait at least 10 min to make sure the cantilever has reached thermal stabilization (lack of further changes in deflection) in the liquid and adjust the vertical and lateral deflection accordingly.

***Note:*** When performing measurements at other temperatures, heat the well to the desired temperature by e.g., a Eurotherm 2216e heating element. Thermal stabilization may take longer in this case and can be confirmed by the absence of further changes in the vertical deflection signal.

**Figure 2:**
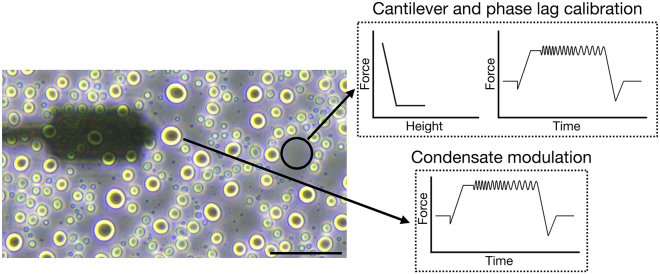
Cantilever calibration and condensate measurements Representative phase-contrast micrograph showing the condensates and the cantilever. The area inside the black circle is free from the condensates and can be used for cantilever as well as phase lag calibration. Scale bar is 50 μm.

**Figure 3:**
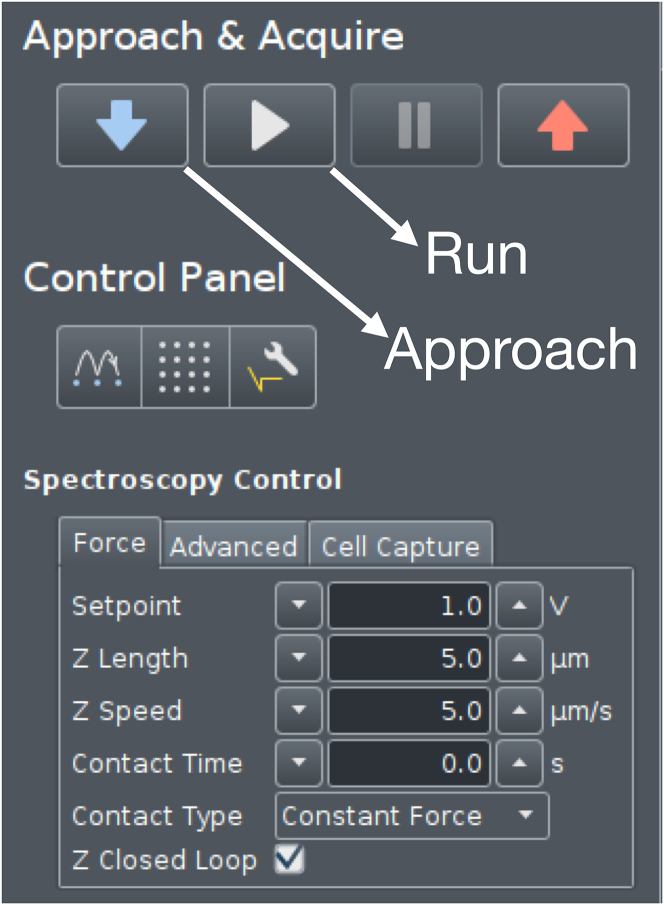
Approach setting Control panel depicting the force settings for the approach (landing the indenter in the surface).

**Figure 4:**
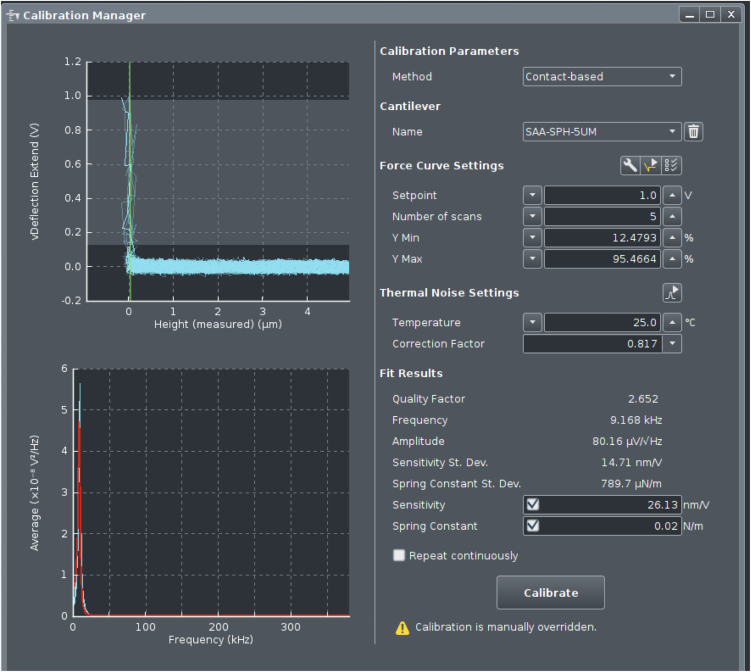
Calibration settings Calibration Manager showing the settings as well as exemplary traces of cantilever calibration.

3. Cantilever calibration.
  a. From the phase contrast live image, choose an area of the dish where no condensates are present and the surface is free (e.g., the area circled in Figure 2).
  b. Move the probe of the cantilever there with the X-Y microscope stage.
  c. Perform an Approach (The blue arrow button in Figure 3) by landing the indenter on the empty area with a setpoint of 1 V.
  d. In the “Acquire Data” tab of the software, choose advanced view and open Calibration Manager (see Figure 4).
  e. Perform a contact-based calibration on the same empty spot of the dish with a setpoint of 1 V, the thermal noise calibration^12^ will automatically follow to deduce both the spring constant and the sensitivity of the cantilever.

***Note:*** Alternatively, if the surface is covered with condensates, or when the probe length is not long enough to ensure a lack of contact with the droplets surrounding the empty area, the calibration can be performed in a separate liquid. In this case in order to keep the concentration of all elements including poly-L-lysine and heparin in the dilute phase the same as the actual experiment, it is recommended to carefully remove the supernatant of another bulk sample and use it for calibration.

***Note:*** For rectangular cantilevers, you can determine both the spring constant and the sensitivity by the contact-free calibration which works according to the principles of calibration based on the thermal noise method.^12^ Here it is critical to specify the cantilever length and width.

***Note:*** For pre-calibrated cantilever, you can specify the spring constant indicated on the cantilever box and only run the thermal noise for the determination of sensitivity.

4. Phase lag calibration.
  a. In Advanced Mode under Spectroscopy Control, create a measurement sequence (Force Ramp Designer) with the following steps.
    i. Force (F) approach.
    ii. Pause.
    iii. 15 height (Z) modulations.
    iv. Height (Z) detach.
  b. Set the parameters as follows:
    i. Setpoint: 1 nN.
    ii. Pause time: 2 s.
    iii. Frequencies: 100, 72, 52, 37, 27, 19, 14, 10, 7.2, 5.2, 3.7, 2.7, 1.9, 1.4, 1 Hz.
    iv. Amplitude: 5 nm.
    v. Z displacement: 10 μm.
  c. Press the play button (see Figure 3) to run the program on the same empty spot of the dish.

***Note:*** The chosen frequencies will lead to equidistant X-axis values in logarithmic representation of the data.

**CRITICAL:** Perform phase lag calibration before each measurement series, especially when changing media or buffer conditions. Fluid properties can alter cantilever drag and influence measurements.

**Figure 5:**
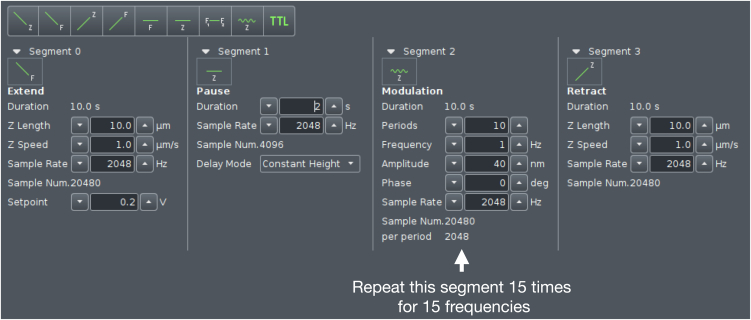
Condensate measurement settings Force Ramp Designer depicting the necessary sequences as well as exemplary settings for the droplet measurements. The Modulation segment needs to be added 15 times in total.

**Figure 6:**
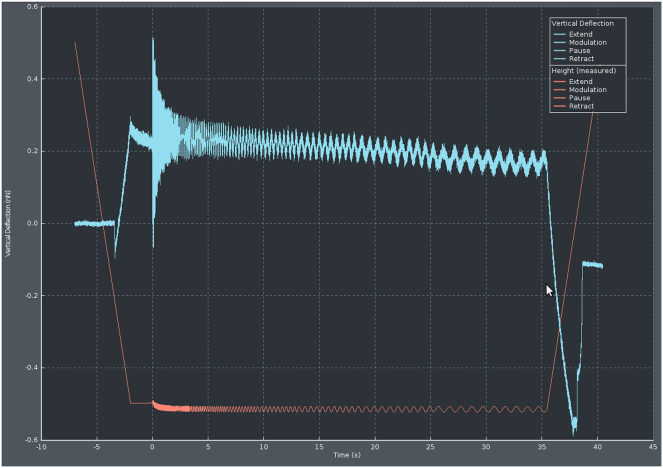
Exemplary force and height traces of condensate measurements Representative vertical deflection (in blue), and height (in red) signals of droplet measurements.

5. Condensate measurements.
  a. After calibrating the cantilever and the phase lag, choose a droplet for measuring and take an image of the droplet to allow determination of radius.
  b. Move the probe of the cantilever in the middle of the condensate with the X-Y microscope stage.
  c. Choose the following parameters in the measurement sequence (Force Ramp Designer) for liquid droplets (see Figure 5):
    i. Setpoint: 0.2 nN.
    ii. Pause time: 2 s.
    iii. Frequencies: 100, 72, 52, 37, 27, 19, 14, 10, 7.2, 5.2, 3.7, 2.7, 1.9, 1.4, 1 Hz.
    iv. Amplitude: 40 nm.
    v. Z displacement: 10 μm.
  d. Run the program by pressing the play button.
  e. Monitor the Force Spectroscopy window in real time to confirm smooth measurement acquisition similar to Figure 6 (troubleshooting 1).
  f. Afterward, move to another droplet by means of the X-Y microscope stage and repeat step 5 of condensate measurements (troubleshooting 2).
  g. Gather all measurements of the same condition to the same folder to facilitate batch processing.

***Note:*** When applying SPM to biomolecular condensates, several considerations are critical. The stiffness of the cantilever defines the accessible force range, and appropriate cantilever selection is essential for reliable data acquisition. In case the stiffness of condensates is unknown, estimation based on the literature or preliminary results may be necessary to choose a cantilever with the right spring constant. In addition, the size of the measured droplets should be taken into consideration so the probe is smaller than the condensate diameter to ensure meaningful contact mechanics. These constraints should be carefully considered when designing experiments (troubleshooting 3).

***Note:*** Since the approach and each modulation frequency are evaluated independently to account for changes in indentation depth, the cantilever drift, visible as a shift in the baseline before indentation and after detachment, does not significantly impact the results.

***Note:*** For harder condensates or condensates after gelation choose larger setpoints of up to 1 nN to achieve sufficient indentation, and reduce the amplitude to as low as 5 nm to avoid too high force signal.

***Note:*** For harder condensates of condensates after gelation, the response of the droplet becomes less dependent on frequency, and the force amplitude remains relatively constant across all frequencies.

***Note:*** Measurement of at least 6–8 condensate droplets and three independent sample preparations is advised. Where high variability is observed, additional replicates should be performed.

### Evaluation procedures

**Timing: 1–2 h**

The following section describes the steps that need to be taken in order to obtain the shear modulus as well as the viscosity of condensates.

**Figure 7:**
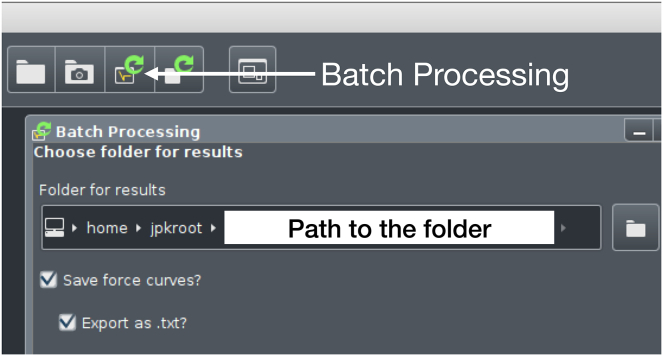
File format adjustment. Batch processing of force files to export them as .txt

6. Determine the radius of the droplet by means of a program such as ImageJ.
7. Using the Batch Processing in the JPK data processing software export the data belonging together as .txt (Figure 7).
8. Make sure to include “Vertical tip position, Vertical deflection, Height, Height (measured and smoothed), Height (measured), Series time, Segment time”.
9. Use the provided Mathematica notebook Biomolecular_Condensate_Rheology_v2.nb.
10. Firstly, obtain the phase calibration data for phase lag removal:
  a. Specify a measurement belonging to the empty surface file name (variable `filename`) relative to the notebook location.
  b. Run the .txt measurement file through Biomolecular_Condensate_Rheology_v2.nb (troubleshooting 4).
  c. Take the list of phi (which is the same as Arg(X)) over frequency (f) and load it into a scientific data analysis tool (e.g., Origin).
  d. Linearly fit the phi(f) data.

***Note:*** It is recommended to average multiple phi(f) data and then fit the average.

11. In order to obtain the complex shear modulus of the condensate:
  a. Put the slope and intercept from the fit of phi(f) into the variables phaseOffset and phaseSlope in Biomolecular_Condensate_Rheology_v2.nb, respectively.
  b. Specify particle radius (variable `particleRadius`).
  c. Enable surface energy compensation (variable `compensateSurfaceEnergy`) for liquid droplets.
  d. Specify touchdown mode (variable `touchdown`):
    i. Touchdown “Snap”: Specify the filter length (variable snapFilterLength), the keepout (variable snapKeepout) and the baseline length (variable snapBaselineLength). Use this in case of interactions between the condensate and the SPM probe before indentation.
    ii. Touchdown “Hard”: Specify the data length (variable hardLength). Use this in case there are no interactions between the condensate and the SPM probe before indentation.
  e. Run the program.
  f. The notebook will produce a file named `G.dat` containing the complex shear modulus as a function of frequency (troubleshooting 5).
  g. The notebook will also generate the surface energy data per measurement file.
12. In order to deduce the viscosity of the condensates, load the G” data in a scientific data analysis tool (e.g., Origin) and perform a linear fit of G″ over frequency. The slope of the fit corresponds to viscosity.

## Expected outcomes

Using this protocol and the associated evaluation notebook, users will be able to quantitatively measure mechanical properties of condensate droplets including viscoelastic rheological characteristics (complex shear modulus), viscosity, as well as surface energy, across liquid-like and gel-like condensates. These measurements will reveal the full spectrum of mechanical maturation, allowing to measure solidification and rejuvenation. This is highly valuable in medical and pharmaceutical contexts, allowing for quantifying pathological gelation and assessing the impact of therapeutics to counteract it.

**Figure 8:**
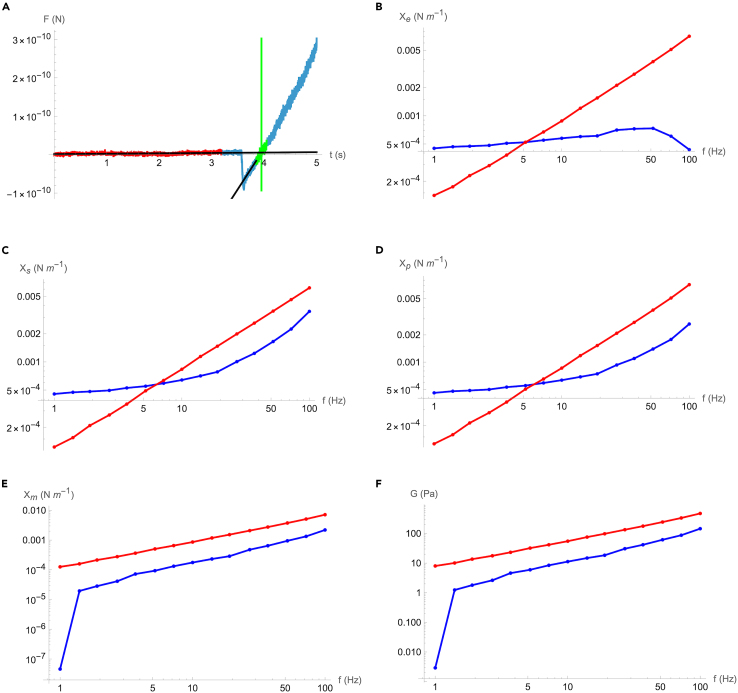
Evaluation steps The data depicted in Figure 6 with the provided Mathematica notebook (See the key resources table) leading to the determination of (A) Force (*F*) vs time (*t*) curves showing condensate indentation with the baseline in red, approximate intersection data points in green, and intersection fits in black and green, (B) Complex experimental spring constant *X*_e_ (C) Complex system spring constant *X*_s_ (D) Complex particle spring constant *X*_*p*_ (E) Complex material spring constant *X*_m_ and (F) Complex shear modulus of condensate *G*. In B-F the blue line is the real (elastic) and the red line is the imaginary (viscous) contribution. Panels B to F depict the sequential data processing steps: phase lag removal (B and C), cantilever spring constant correction (C and D), surface energy subtraction (D and E), and finally application of the Hertzian contact model (E and F).

The evaluation of the data presented in Figure 6 with the provided Mathematica analysis notebook, yields the results shown in Figure 8. The full details on the evaluation steps and the assays developed are described in the primary research article.^1^ Here, the first step involves identifying the contact point, where the onset of indentation of the condensate by the cantilever probe is determined. This step is necessary to account for the abrupt contact between the indenter and the droplet, detectible as a sharp drop in force (Figure 8A).

Subsequently, the frequency-dependent response of the system is evaluated. For this the force and height modulation amplitudes are fitted with a sinusoidal function at each excitation frequency.^13^ This leads to the determination of the complex experimental spring constant *X*_e_ (Figure 8B).

To isolate the viscoelastic response of the sample, the phase lag caused by the hydrodynamic drag of the cantilever in the liquid environment and measured as described in “*Phase lag calibration*” is removed.^14^ The result is the complex system spring constant *X*_s_ (Figure 8C).

Next, the contribution of the cantilever, which is determined by calibrating it in section “*Cantilever calibration*”, and is in a series connection with the particle’s spring constant, is subtracted. This allows to obtain the effective particle spring constant of the condensate *X*_p_ (Figure 8D).

The following step removes the influence of surface energy, which acts as a parallel connection with the bulk material spring constant *X*_*m*_ (Figure 8E).^13^^,^^15^^,^^16^ Once the bulk spring constant is isolated, the final step applies the Hertzian contact model, which describes the deformation of two solid curved bodies.^17^^,^^18^ With this, the material spring constant can be translated into the frequency-dependent complex shear modulus of the condensate *G* (Figure 8F). This modulus captures both the storage (elastic) and loss (viscous) components of the condensate material under dynamic deformation.

**Figure 9:**
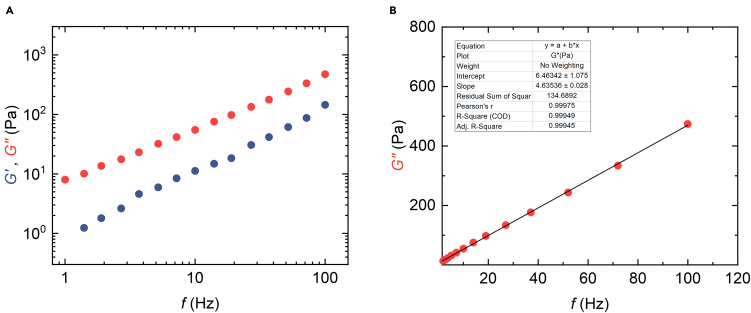
Plotting the data and evaluation of viscosity The plotted shear modulus *G* data from Figure 8 showing (A) The elastic (*G*′, blue) and viscous (*G*″, red) shear moduli of condensates (B) Linear fit of the of *G*″ for determination of viscosity.

The G.dat file generated by the Mathematica notebook can be further processed by plotting the data and fitting the G″ linearly over frequency to achieve viscosity as depicted in Figure 9. The notebook also outputs the surface energy values.

***Note:*** Robust measurements can be achieved relatively quickly by users with general experience in SPM and atomic force microscopy. However, if the instrument is operated by new users or if entirely unknown condensates are being measured, where no literature values are available for comparison, the viscosity data obtained via SPM can be validated using complementary techniques such as fluorescence recovery after photobleaching, as outlined in the primary research paper.^1^

## Limitations

A limitation of SPM-based measurements is that condensates must adhere sufficiently to the substrate. Without adequate attachment to the bottom of the dish, droplets may shift or slide under the cantilever during indentation. Adjustments to the surface passivation may be necessary to counteract this limitation.

The Hertzian contact model assumes that the condensate is homogeneous and isotropic. However, this assumption may not hold for all droplet chemistries. Some protein condensates exhibit spatial heterogeneity, such as solidification initiating at the surface and progressing inward.^19^ In such cases, measurements should only be performed after full solidification to ensure that the derived rheological parameters reflect a uniform material phase.

The application of the Hertzian contact model in our analysis requires specific assumptions about droplet geometry: the single Hertzian contact model is suitable for hemispherical condensates, and the double Hertzian contact model is applicable to spherical droplets.^1^ Consequently, droplets must be approximated as either hemispherical or spherical. Intermediate or irregular shapes cannot be fully accurately described by these models.

SPM is restricted to assessing mechanical properties at the sample surface. As a result, condensates can only be directly measured *in vitro*, at plasma membrane,^5^ or extracellularly while interacting with cells.^4^

## Troubleshooting

### Problem 1

• Non-specific interactions between the cantilever and condensate droplet (related to Step 5e in SPM measurement).
• Non-specific interactions such as electrostatic forces may look like abrupt or adhesive contact between the cantilever and the condensate before indenting the droplets.

### Potential solution

To reduce such interactions, recoat the cantilever with Pluronic F-127, which has been previously reported as an effective passivation strategy for studying condensates.^11^ In case residual interactions still occur, refine the point-of-contact (Touchdown) determination to snap in the provided evaluation notebook to account for these effects and allow for determination of the unperturbed geometry of the condensate.

### Problem 2

• Condensates evaporate during measurements (related to Step 5f in SPM measurement).
• While the well where condensates are made can be sealed using a silicone ring around the cantilever holder, this does not provide a fully airtight seal. During prolonged measurements, condensates may undergo evaporation, which can affect their mechanical properties and measurement accuracy.

### Potential solution

To avoid evaporation, condensates can be covered with a layer of mineral oil (e.g., Sigma-Aldrich product no. 330779). Note that both the cantilever and the glass holder will come into contact with the oil. It is therefore essential to thoroughly clean these components after use to prevent residue buildup or contamination of future samples. It is recommended to consult the SPM manufacturer for the appropriate cleaning procedure tailored to your specific instrument.

### Problem 3

• Cantilever is too stiff or too soft for probing condensates (related to Step 5 in SPM measurement).
• If the cantilever is too stiff, it may not deform sufficiently upon contacting the condensate, resulting in poor sensitivity and inaccurate force measurements. Conversely, if the cantilever is too soft, thermal noise and instability can reduce measurement precision.

### Potential solution

Select a cantilever with an appropriate spring constant based on the expected stiffness of the condensate or with preliminary experiments.

### Problem 4

• Droplets are prepared on a soft surface (related to 10b in evaluation procedures).
• In case condensates are placed on a soft surface such as living cells, the assumption of the Hertzian contact model that the substrate is infinitely stiffer than the sample no longer holds and the substrate may also deform under the cantilever force.

### Potential solution

First, use the same protocol described here to independently measure the average rheological properties of the soft substrate (e.g., a cell). Then, apply the stacked Hertzian model developed by Naghilou et al.,^4^ which takes the deformation and viscoelastic properties of the underlying soft substrate under consideration to extract the condensate mechanical properties.

### Problem 5

Low signal-to-noise ratio at low-frequency modulation (related to Step 11f in evaluation procedures) At low-frequency modulation, force-distance curves can exhibit a poor signal-to-noise ratio, especially in soft, dynamic condensates. This noise can propagate through the analysis workflow into calculations of surface energy and ultimately compromise the reliability of derived shear modulus values.

### Potential solution

Use the quasi-static indentation evaluation approach. Instead of relying on modulated data, fit the indentation portion of the force curve directly to determine the surface energy (Equation 9 in the primary research paper^1^). This provides a more accurate and reproducible readout for subsequent mechanical parameter extraction.

## Resource availability

### Lead contact

Requests for further information should be directed to and will be fulfilled by the lead contact, Alireza Mashaghi (a.mashaghi.tabari@lacdr.leidenuniv.nl).

### Technical contact

Further information and requests about the technical specifics of performing the protocol should be directed to and will be fulfilled by the lead technical contact and first author, Aida Naghilou (a.naghilouye.hidaji@lacdr.leidenuniv.nl or aida.naghilou@meduniwien.ac.at).

### Materials availability

This paper did not generate any new reagents. All materials used can be purchased from the manufacturers.

### Data and code availability

The code and data discussed in this protocol are available in the supporting information of the primary research paper^1^ and the associated Zenodo and GitHub repositories.

## Acknowledgments

The authors thank Vahid Sheikhhassani and Oskar Armbruster for critical reading of this article. The graphical abstract includes an element adapted from Naghilou et al.,^1^ Cell Reports Physical Science (2025), published under a CC BY license. This project was partly supported by the Dutch Research Council (NWO), Open Competition grants OCENW.XS23.3.105 and OCENW.XS22.4.185.

## Author contributions

A.N.: conceptualization, methodology, formal analysis, investigation, writing – original draft, writing –review and editing, and visualization. A.M.: conceptualization, methodology, writing – review and editing, supervision, project administration, and funding acquisition.

## Declaration of interests

The authors declare no competing interests.
